# Surgery for Retroperitoneal Soft Tissue Sarcoma is Safe Following Multimodal Treatment with Regional Hyperthermia

**DOI:** 10.1245/s10434-025-18141-8

**Published:** 2025-08-29

**Authors:** Mathilda Knoblauch, Cathy Werdel, Yanik Arlt, Lars H. Lindner, Dorit Di Gioia, Anton Burkhard-Meier, Luc Berclaz, Nina-Sophie Schmidt-Hegemann, Paul Schmidt, Jens Werner, Markus Albertsmeier

**Affiliations:** 1 Department of General, Visceral and Transplantation Surgery, Ludwig Maximilians Universität (LMU) Munich, LMU Hospital, Munich, Germany; 2 Department of Medical Oncology, Ludwig Maximilians Universität (LMU) Munich, LMU Hospital, Munich, Germany; 3 Department of Radiation Oncology, Ludwig Maximilians Universität (LMU) Munich, LMU Hospital, Munich, Germany; 4 Statistical Consulting for Science and Research, Berlin, Germany

**Keywords:** Retroperitoneal neoplasms, Sarcoma, Neoadjuvant therapy, Radiotherapy, Adjuvant, Drug therapy, Hyperthermia, Induced, Postoperative complications, Surgical procedures, Operative, Morbidity, Neoplasm recurrence, Local

## Abstract

**Background:**

Multimodal treatment has been suggested to improve outcomes in retroperitoneal soft tissue sarcoma (RPS). This study assessed the impact of neoadjuvant radiotherapy, chemotherapy, and the combination with regional hyperthermia (RHT) on postoperative morbidity using the Comprehensive Complication Index (CCI).

**Patients and Methods:**

This single-center retrospective cohort study analyzed 335 surgeries for primary and recurrent RPS (2009–2022). The primary outcome was CCI, which was modeled using zero-one inflated beta regression. Predictors included age, sex, recurrence, transfusion needs, and the number of resected organs.

**Results:**

Patients with neoadjuvant radiotherapy (CCI, 32.79 ± 25.24 vs 22.76 ± 26.75), neoadjuvant chemotherapy (28.06 ± 24.35 vs 23.55 ± 27.75), and the combination of neoadjuvant chemotherapy + RHT (31.52 ± 25.78 vs 22.54 ± 26.42) had higher CCI scores compared with patients without these treatments. In multivariable models of the probability of a complication (CCI > 0) or of the severity (0 < CCI < 100) these neoadjuvant treatments did not significantly influence CCI. The resection of > 3 organs was associated with more frequent (*p* = 0.004) and more severe (*p* = 0.002) complications, as were blood transfusions (CCI > 0, *p* = 0.030; 0 < CCI < 100, *p* = 0.034). When the interaction between therapy and recurrence was added to these models, the combination of radiotherapy and chemotherapy (without RHT, *n* = 7) was associated with higher morbidity, whereas all other protocols appeared to be equally safe in primary and recurrent cases.

**Conclusions:**

Postoperative morbidity is driven by surgical factors, particularly transfusions and multivisceral resections, rather than multimodal neoadjuvant therapies. Intensive protocols, including RHT, are safe for patients with RPS from a surgical perspective.

**Supplementary Information:**

The online version contains supplementary material available at 10.1245/s10434-025-18141-8.

Soft tissue sarcomas are a rare group of malignant tumors originating from mesenchymal cells. They are most commonly found in the extremities but develop in the retroperitoneum in about 15% of cases.^1^ While radical surgery remains the only curative option for localized disease, the effectiveness of perioperative oncological treatments continues to be a topic of scientific debate. Different histological subtypes of retroperitoneal sarcoma show characteristic behaviors regarding the development of local recurrence or distant metastases, suggesting that the indication for perioperative treatment should be based on histology and grading, among other factors.^2^ The recent STREXIT study pooled data from the randomized controlled STRASS trial^3^ with matched off-study patients, indicating that radiotherapy (RT) may be effective in the group of well-differentiated liposarcoma (WDLPS) and G1/G2 dedifferentiated liposarcoma (DDLPS) but not in G3 DDLPS or leiomyosarcoma (LMS). The ISG-STS 1001 trial showed that histotype-tailored neoadjuvant chemotherapy (CTx) was inferior to standard anthracycline-based treatment for high-risk extremity and trunk wall sarcomas, supporting preoperative anthracycline-based therapy in patients with a high probability of tumor progression.^4^ For retroperitoneal tumors, neoadjuvant systemic therapy is currently being investigated in the STRASS 2 trial.^5^ Finally, the EORTC 62961-ESHO 95 randomized clinical trial showed improved local recurrence-free, disease-free, and overall survival in high-risk patients treated with regional hyperthermia (RHT) in addition to chemotherapy.^6^ RHT involves deep-tissue heating of the tumor region using an external applicator and has been suggested to enhance cytotoxicity and the peritumoral immune response.^7,8^

In conclusion, patients with high-risk RPS may be eligible for different neoadjuvant treatment protocols depending on tumor and patient characteristics, and some patients will receive multimodal treatment combining two or three of the above modalities. The toxicity of neoadjuvant therapies has been described as acceptable in clinical trials,^3,4,6^ and it was not a significant risk factor in a retrospective analysis of perioperative morbidity by the Transatlantic Australasian Retroperitoneal Sarcoma Working Group (TARPSWG).^9^ However, this analysis excluded all nonsevere complications (Clavien–Dindo I–II)^10^ and did not further differentiate the severity of morbidity. Furthermore, the influence of neoadjuvant RHT on postoperative complications has not been examined.

Therefore, we designed this retrospective study to determine the influence of multimodal therapies, including RHT, on perioperative morbidity in patients with primary and recurrent RPS using the Comprehensive Complication Index (CCI)^11^ as the primary outcome parameter. The CCI is based on the Clavien–Dindo classification^10^ and yields a weighted sum score between 0 (no complications) and 100 (death) to comprehensively assess postoperative morbidity in a given patient. Hence, a preliminary aim of this study was to develop statistical models for the multivariable analysis of the CCI.

## Patients and Methods

### Study Design and Setting

This single-center retrospective cohort study aimed to investigate the influence of neoadjuvant multimodal therapies on perioperative morbidity in primary and recurrent RPS. Between January 2009 and December 2022, adult patients undergoing curative-intent surgery for RPS were recruited at Ludwig Maximilians Universität (LMU) Hospital, a high-volume sarcoma center. Two separate patient cohorts were formed for primary and recurrent tumors. Follow-up for survival was continued until December 2022. The LMU Ethics Committee approved the study protocol (ref. 23-0067).

### Participants

Patients were included if primary or subsequent surgeries for RPS were performed at LMU Hospital during the study period. Patients with Ewing sarcoma, rhabdomyosarcomas, desmoid tumors, gynecological sarcomas, and gastrointestinal stromal tumors were excluded. Patients were followed in outpatient clinics of the medical oncology, radiation oncology, and surgery departments. The study cohort included patients who received neoadjuvant chemotherapy alone or combined with regional hyperthermia, with or without additional radiotherapy, as well as patients who received no neoadjuvant treatment.

### Interventions

Perioperative treatment decisions were made by a multidisciplinary tumor board. During the study period, eligible patients with high-risk tumors (G2/G3, > 5 cm) were generally offered multimodal treatment, including a combination of anthracycline-based neoadjuvant chemotherapy and RHT. Regional hyperthermia was applied during chemotherapy infusions on days 1 and 4 of each cycle and consisted of regional deep-tissue heating using a capacitive phased-array system. A small number of patients unfit for multimodal treatment received chemotherapy alone. Typically, four cycles of chemotherapy combined with RHT were administered preoperatively, followed by an additional four cycles postoperatively.

Radiotherapy was applied in patients with a high expected risk of local recurrence. Patients with low-risk tumors and patients who could not receive either effective systemic treatment or radiotherapy were typically managed with upfront surgery.

### Outcomes

The main outcome of the study was the CCI, as described by Slankamenac et al.^11^ It was calculated using the spreadsheet tool provided by the authors, which generates a continuous score from 0 (no complications) to 100 (death), on the basis of Clavien–Dindo classification of all postoperative complications.^10^ By summing all complications and weighting them according to severity, the CCI provides a more nuanced measure of cumulative morbidity than traditional endpoints that consider only the presence or the most severe complication. Although originally developed in prospective trials, the CCI can be reliably applied in retrospective studies provided that complication data are systematically recorded.^12^ In our setting, structured electronic perioperative documentation enabled consistent retrospective classification and scoring.

Overall survival and local recurrence-free survival were secondary outcomes. A variety of predictors were included in the analysis, such as neoadjuvant treatment protocols, age, sex, number and type of resected organs, transfusion requirements, and the number of recurrences.

### Data Sources

Data were retrieved retrospectively from electronic patient records at LMU Hospital. From April 2017 onward, data were collected in a prospective database (ref. 768-16; LMU Ethics Committee). Survival data were cross-checked using the Munich Online Comprehensive Cancer Analysis Platform.^13^

### Bias

To minimize selection bias, the study included all documented consecutive patients who met simple but strict inclusion and exclusion criteria, ensuring that participants were selected on the basis of predefined inclusion criteria rather than the selection being influenced by predictors or outcomes. Information bias was addressed by starting recruitment in 2009, when more reliable patient data were available compared with previous times. The validated CCI was used to enhance the accuracy of the primary outcome parameters. Multiple data sources were cross-checked when they were available. Lastly, potential confounders were identified and measured, and statistical techniques, such as multivariable analysis, were employed to adjust for confounding variables and isolate the effect of exposure on the outcome of interest.

### Statistics

The patient cohort was described using rates and proportions for categorical variables, and means with standard deviations for continuous variables, as appropriate. For univariable analysis of morbidity, mean CCI values were compared graphically between groups using box plots.

The distribution of the CCI values is a two-sided truncated distribution. An analysis of these values using standard methods (i.e., linear models) would entail a high potential for bias. Therefore, we used zero-one inflated beta regression models for the multivariable analysis of CCI values. In the first step, the CCI is divided by 100 to fit within the interval [0,1]. The model comprises three components: one to estimate the probability of any complication (CCI > 0), one to describe the severity of complications (0 < CCI < 100), and one to capture the probability of death (CCI = 100). Because death was only rarely observed, the potential bias introduced by these values can be considered low. Therefore, the last model component was omitted, which reduced the model to a zero-inflated beta model. Consequently, values of CCI = 100 were replaced with 99.99 and modeled using the 0 < CCI < 100 component.

Neoadjuvant therapies, sex, age, perioperative transfusion requirements, the number of resected organs, and number of recurrences were included as fixed effects. Therapeutic approaches were categorized into five groups: no therapy, chemotherapy, radiotherapy, chemotherapy combined with RHT, and chemotherapy combined with RHT and radiotherapy. For age, a cutoff of 70 years was chosen in line with standard clinical practice, and it was additionally modeled as a continuous variable, whereas sex was represented as a binary characteristic. The total number of resected organs was dichotomized with a cutoff of < 3 versus ≥ 3 organs on the basis of findings reported by Bonvalot et al.^14^ In addition, the number of resected organs was modeled as a continuous predictor. Although prior research suggests that the influence of the number of removed organs may be nonlinear,^14^ this was not addressed separately, as the inherent flexibility of the beta regression model adequately captures nonlinear relationships. The number of recurrences was included as a continuous variable and as primary versus recurrent tumors. In an exploratory analysis, grading did not significantly influence morbidity and was therefore not included in the model.

Incorporating information about the removed organs adds further complexity. Because of the relatively large number of potential organs and the variable influence of the total number of resected organs, modeling this information as random effects was deemed most appropriate. Furthermore, since multiple organs could be removed simultaneously, this information was modeled using a multi-membership grouping approach to account for overlapping contributions.

To determine whether the effects of therapy differed between patients with and without recurrence, an additional model incorporating the interaction between therapy and recurrence was estimated.

Missing data were not included in the calculations unless otherwise specified; the number of patients with missing data is reported in the tables. All analyses were conducted using R version 4.2.2.^15^

## Results

### Patients

We analyzed 335 consecutive curative-intent surgeries. Table 1 presents the clinicopathologic characteristics of the study cohort separately for the whole cohort and the three major treatment groups (no therapy, CTx + RHT, and CTx + RHT + RT). Owing to their infrequent application, CTx alone, RT alone, and the combination of CTx and RT are not shown. The mean age at the time of resection was 59.1 ± 12.8 years. Of these, 183 (55%) were male, and 152 (45%) were female. A total of 153 patients underwent surgery for primary tumors, while 182 procedures were performed for recurrent tumors. The number of prior recurrences among patients ranged from one to eight, with 82 (45.1%) experiencing one recurrence, 45 (24.7%) two recurrences, 18 (9.9%) three, 15 (8.2%) four, 13 (7.1%) five, 5 (2.7%) six, and 2 (1.1%) each with seven and eight recurrences. We here report the analysis of the full study population; subgroup-specific data for patients with primary and recurrent tumors are provided in the Supplemental Materials.

**Table 1:** Baseline clinicopathologic characteristics of the study cohort

Characteristics	All	No therapy	CTx + RHT	CTx + RHT + RT
	(*n* = 335)	(*n* = 210)	(*n* = 49)	(*n* = 36)
Sex, *n* (%)				
Female	152 (45)	96 (45.7)	28 (57.1)	12 (33.3)
Male	183 (55)	114 (54.3)	21 (42.9)	24 (66.7)
Age, mean (± SD), years	59.1 (± 12.4)	58.8 (± 13.2)	55.8 (± 13.2)	60.4 (± 11.1)
Histological subtype, *n* (%)				
Well-differentiated liposarcoma	64 (19.1)	51 (24.3)	4 (8.2)	3 (8.3)
Dedifferentiated liposarcoma	146 (43.6)	81 (38.6)	22 (44.9)	22 (61.1)
Leiomyosarcoma	37 (11)	19 (9.0)	10 (20.4)	3 (8.3)
Undifferentiated pleomorphic sarcoma	54 (16.1)	36 (17.1)	9 (18.4)	6 (16.6)
Other	34 (10.5)	23 (11.0)	4 (8.2)	2 (5.6)
Grading, *n* (%)				
G1	69 (20.6)	54 (25.7)	4 (8.2)	4 (11.1)
G2	119 (35.5)	66 (31.4)	16 (32.7)	20 (55.6)
G3	118 (35.2)	66 (31.4)	27 (55.1)	10 (27.8)
Missing	29 (8.7)	24 (11.4)	2 (4.1)	2 (5.6)
Blood transfusion (± SD), mL	255.8 (± 613.3)	176.2 (± 485.8)	393.5 (± 720.7)	408.3 (± 895.7)
R status, *n* (%)				
0	159 (47.5)	82 (39.0)	26 (53.1)	24 (66.7)
1	119 (35.5)	82 (39.0)	15 (30.6)	10 (27.8)
2	40 (11.9)	31 (14.8)	8 (16.3)	2 (5.6)
x	1 (0.3)	0 (0)	0 (0)	0 (0)
Missing	16 (4.8)	15 (7.1)	0 ()	0 (0)
Neoadjuvant radiation therapy, *n* (%)				
Yes	65 (19.4)			
No	265 (79.1)			
Missing	5 (1.5)			
Neoadjuvant chemotherapy, *n* (%)				
Yes	104 (31)			
No	225 (67.2)			
Missing	6 (1.8)			
Neoadjuvant hyperthermia, *n* (%)				
Yes	90 (26.9)			
No	224 (66.9)			
Missing	21 (6.3)			

The cohort included 64 patients with well-differentiated liposarcoma (WDLPS, 19.1%), 146 with dedifferentiated liposarcoma (DDLPS, 43.6%), 37 with leiomyosarcoma (LMS, 11%), 54 with undifferentiated pleomorphic sarcoma (UPS, 16.1%), and 34 with other types of sarcoma (10.5%). Tumors were classified according to the Fédération Nationale des Centres de Lutte Contre le Cancer (FNCLCC) grades as follows: 69 tumors (19.9%) were grade 1, 119 (35.5%) were grade 2, and 118 (35.2%) were grade 3.

The mean operation time was 262.6 ± 118.6 min, and patients received an average of 255.8 ± 613.3 mL of red blood cell transfusions. Resection margins were R0 in 159 patients (47.5%), R1 in 119 patients (35.5%), and R2 (including tumor perforations) in 40 patients (11.9%).

### Treatments

In total, 210 patients (62.7%) underwent resection without neoadjuvant treatment. Neoadjuvant chemotherapy was administered as the only preoperative treatment in 11 (3.3%) patients. It was combined with radiotherapy in 7 patients (2.1%), with RHT in 49 patients (14.6%), and with both radiotherapy and RHT in 36 patients (10.8%), adding to a total of 103 patients who received neoadjuvant systemic treatment. Neoadjuvant radiotherapy was administered to 62 patients (18.7%); this was the only neoadjuvant treatment in 19 of these patients (5.7%). Treatment information was missing for three patients.

### Postoperative Complications

The Comprehensive Complication Index (CCI) for all patients with available data (*n* = 269) was 25.01 ± 26.43 (mean ± standard deviation (SD)). The distribution of complications according to the Clavien–Dindo classification was as follows: 107 patients (31.9%) had no complication, 32 (9.6%) had grade 1, 52 (15.5%) had grade 2, 113 (33.7%) had grade 3, 10 (3.0%) had grade 4, and 12 patients (3.6%) died within 30 days. Reoperations were required in 77 patients (23.0%), and interventional drainage placement was necessary in 54 patients (16.1%) (Table 2).

**Table 2:** Postoperative complications of the study cohort

Characteristics	All
The Comprehensive Complication Index, (± SD)	25.01 (± 26.53)
Clavien–Dindo, *n* (%)	
Grade 0	107 (31.9)
Grade 1	32 (9.6)
Grade 2	52 (15.5)
Grade 3	113 (33.7)
Grade 4	10 (3.0)
Grade 5	12 (3.6)
Missing	9 (2.7)
Mortality, *n* (%)	
Yes	15 (4.3)
No	318 (94.9)
Missing	2 (0.6)
Reoperation, *n* (%)	
Yes	77 (23.0)
No	250 (74.6)
Missing	8 (2.4)
Postoperative drainage, *n* (%)	
Yes	54 (16.1)
No	275 (82.1)
Missing	6 (1.8)

### Univariate Analysis of Postoperative Complications

**Fig. 1:**
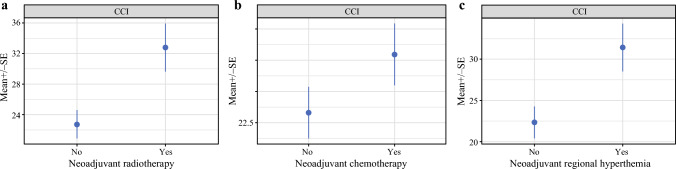
Comprehensive Complication Index following neoadjuvant radiotherapy (**A**); chemotherapy with or without regional hyperthermia (**B**); and chemotherapy combined with regional hyperthermia (**C**)

On univariate analysis, complications were more frequent and/or severe in patients who had received neoadjuvant treatment. In detail, patients who received radiotherapy, either alone or combined with chemotherapy ± RHT, had a mean CCI of 32.79 ± 25.24, compared with 22.76 ± 26.75 in those who did not receive radiotherapy (Fig. 1a). In the radiotherapy group, 53.2% of patients experienced postoperative complications ≥ Clavien–Dindo grade 3 compared with 39.0% without therapy. The reoperation rate was 34.4% versus 20.9%, and postoperative drains were required in 23.4% and 15.0% of the patients, respectively.

Among patients receiving neoadjuvant chemotherapy, alone or combined with other treatments, the mean CCI was 28.06 ± 24.35, compared with 23.55 ± 27.75 in those who did not receive chemotherapy (Fig. 1b). After neoadjuvant systemic therapy, 49.1% of the patients had postoperative complications ≥ grade 3 compared with 38.4% in those without. Reoperations were needed in 31.7% versus 20.0% of patients, and postoperative drains were placed in 19.8% versus 15.3% of patients.

**Fig. 2:**
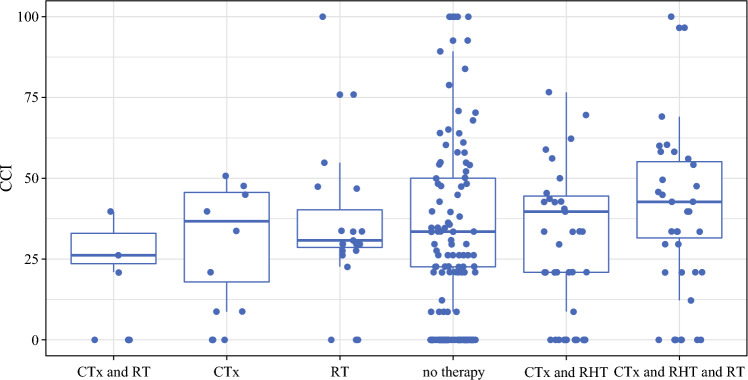
Comprehensive Complication Index following different neoadjuvant treatment protocols

Patients who received RHT showed a mean CCI of 31.52 ± 25.78, compared with 22.54 ± 26.42 in patients without RHT in their neoadjuvant therapy (Fig. 1c). Figure 2 summarizes the CCI for patients undergoing different treatment modalities.

### Multivariate Analysis of Postoperative Complications

**Fig. 3:**
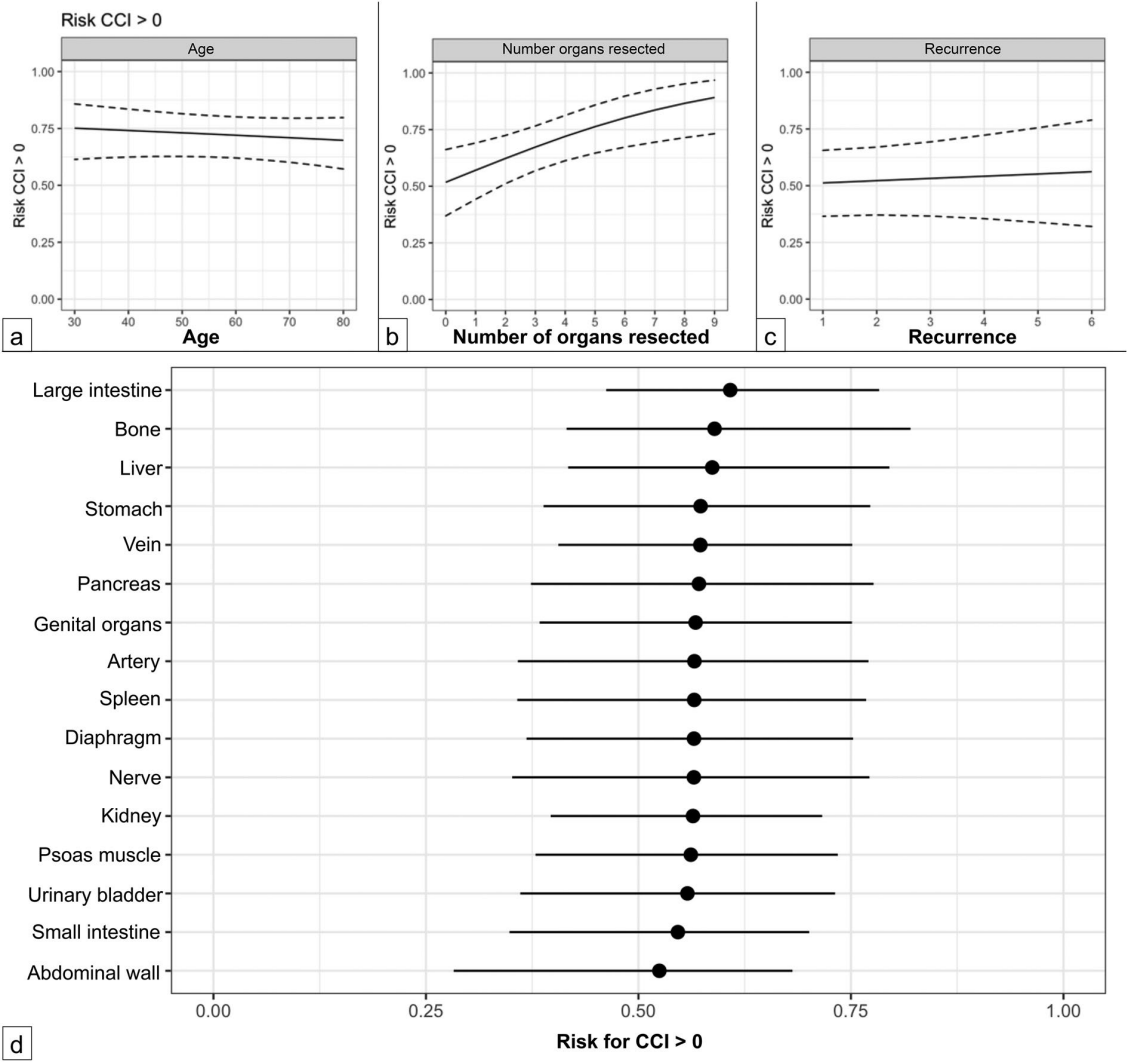
Influence of age (**A**); number of resected organs (**B**), recurrence (**C**); and certain resected organs (**D**) on CCI in the model examining the probability of experiencing any postoperative complication (CCI > 0)

To determine whether neoadjuvant therapies independently influence postoperative morbidity and to identify additional contributing factors, zero-one inflated beta models were constructed as described above. In the model examining the probability of experiencing any postoperative complication (CCI > 0), the need for a blood transfusion (*p* = 0.030) and the resection of three or more organs (*p* = 0.004) were identified as significant factors associated with the occurrence of postoperative complications. Conversely, no correlation was found between the CCI and age, sex, surgery for recurrence, or preoperative treatment (Table 3). Figure 3 illustrates the relationship between the CCI and variables such as age (Fig. 3a), the number of resected organs (Fig. 3b), the number of recurrences (Fig. 3c), and specific resected organs (Fig. 3d).

**Table 3:** Influence of gender, transfusion, age, number of resected organs, and recurrence on CCI in the model examining the probability of experiencing any postoperative complication (CCI > 0)

	Risk CCI > 0 (mean, 95% CI)
Sex	
Female (*n* = 114)	0.73 (0.62–0.82)
Male (*n* = 147)	0.72 (0.6–0.81)
*P* value	0.77
Transfusion	
No (*n* = 132)	0.69 (0.58–0.79)
Yes (*n* = 53)	0.83 (0.71–0.91)
*P* value	**0.03**
Age (years)	
< 70	0.73 (0.63–0.81)
> 70	0.7 (0.58–0.8)
*P* value	0.49
Number of resected organs	
< 3	0.57 (0.44–0.69)
> 3	0.79 (0.62–0.88)
*P* value	**0.004**
Recurrence	
No	0.72 (0.62–0.8)
Yes	0.74 (0.59–0.86)
*P* value	0.65

**Fig. 4:**
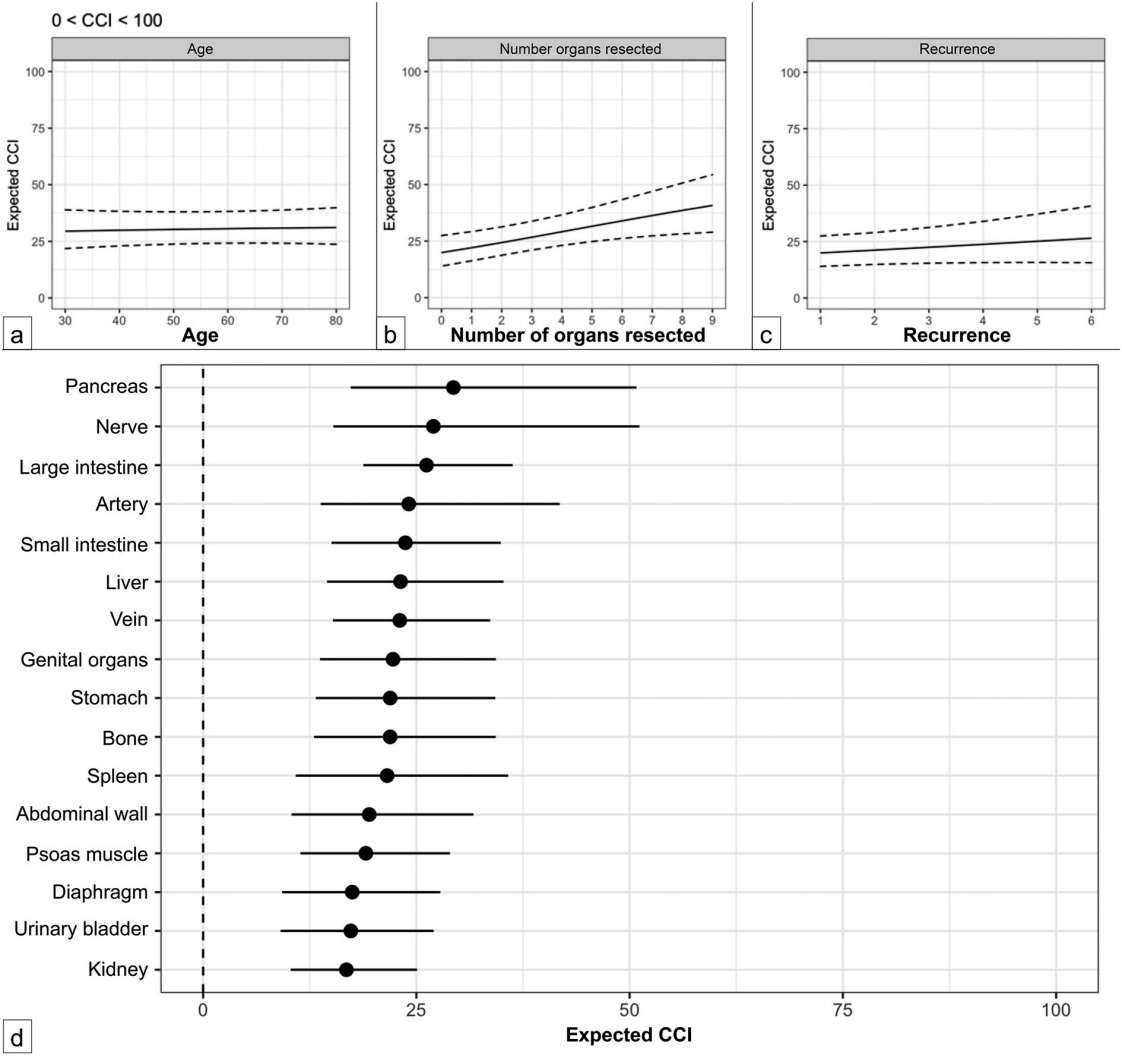
Influence of age (**A**); number of resected organs (**B**), recurrence (**C**); and certain resected organs (**D**) on CCI in the model examining the severity of complications (0 < CCI < 100)

In the model assessing the severity of complications (0 < CCI < 100), both the need for a blood transfusion (*p* = 0.034) and resection of three or more organs (*p* = 0.002) were significantly associated with an increase in the CCI. Again, age, sex, surgery for recurrence, and preoperative treatment did not show any significant correlation with the CCI (Table 4). Figure 4a depicts the relationships between the CCI and factors such as age, Fig. 4b number of resected organs, and Fig. 4c number of recurrences.

**Table 4:** Influence of gender, transfusion, age, number of resected organs, and recurrence on CCI in the model examining the probability of experiencing the severity of postoperative complication (0 < CCI < 100)

	Expected CCI (mean, 95% CI)
Sex	
Female (*n* = 114)	30.6 (24–38.6)
Male (*n* = 147)	30.5 (23.8–38.7)
*P* value	0.99
Transfusion	
No (*n* = 132)	29.3 (22.6–37.1)
Yes (*n* = 53)	36.8 (28.9–46.5)
*P* value	**0.03**
Age (years)	
< 70	30.2 (23.9–38)
> 70	31.1 (23.9–39.6)
*P* value	0.83
Number of resected organs	
< 3	22.2 (16.5–29.3)
> 3	34.1 (26.4–43.3)
*P* value	**0.002**
Recurrence	
No	30.5 (24.3–38.2)
Yes	35.8 (25.9–47.1)
*P* value	0.13

When the interaction between therapy and recurrence was added to these models, the combination of radiotherapy and chemotherapy (without RHT, *n* = 7) was associated with a higher probability of complications (CCI > 0, *p* < 0.001) and higher CCI values (0 < CCI < 1, *p* = 0.010) following the resection of primary tumors (*n* = 5) compared with recurrent tumors (*n* = 2). No such effect was observed for other treatment modalities or combinations.

### Transfusion Requirement and Preoperative Anemia

The need for intraoperative blood transfusion was further analyzed by binary logistic regression, showing that preoperative hemoglobin (Hb) was a significant predictor of the likelihood of requiring a transfusion. For every 1 g/dL increase in preoperative Hb level, the risk of needing a transfusion decreased significantly by 35.6%. Although the model shows an improvement compared with the null model, it explains only a moderate proportion of the variance (Cox and Snell *R*^2^ = 12.9%, Nagelkerke *R*^2^ = 18.5%).

Preoperative hemoglobin levels were then analyzed using multiple linear regression with treatment modalities and the interaction between chemotherapy and RHT as independent factors. In this model, neoadjuvant chemotherapy decreased preoperative hemoglobin levels by 1.8 g/dL (*p* < 0.001). No significant influence was observed for radiotherapy, RHT, or the interaction between chemotherapy and RHT.

## Discussion

This retrospective cohort study found no influence of neoadjuvant multimodal therapies, including regional hyperthermia, on perioperative morbidity in retroperitoneal soft-tissue sarcomas. Postoperative complications were associated with surgical factors, such as transfusion requirements and the extent of surgery.

This study has several limitations. The nonrandom allocation of neoadjuvant treatments may have caused selection bias. Our center treats many high-risk (G2/G3) patients, which may have resulted in an over-representation of aggressive tumors and an overall increase in perioperative morbidity in this cohort. A few patients may not have received their allocated neoadjuvant treatment owing to pre-existing comorbidities, while at the same time being at risk of perioperative morbidity. As much as this effect depends on patient age, it has been included in our beta model; however, an independent effect of comorbidities would result in the underestimation of the negative impact of intensive neoadjuvant treatments.

The completeness of the recorded data improved during the study period, particularly for blood transfusions and Clavien–Dindo grade 1/2 complications. The presence of information bias, although mitigated by the exclusion of patients who underwent surgery before 2009, may have reduced the magnitude of the observed associations.

The integration of confounding variables into the beta model is a strength of this study. One factor that may have influenced both transfusion requirements and morbidity is preoperative anemia, which was further investigated using linear regression models. We found a strong association between neoadjuvant chemotherapy and preoperative anemia, which, in some cases, translated into a need for intraoperative red blood cell transfusion. However, low preoperative hemoglobin levels accounted for only a small portion of red blood cell transfusions; therefore, intraoperative blood loss is likely more important.

This is the first study to examine the influence of multimodal neoadjuvant therapy with RHT on perioperative morbidity in patients with RPS. Following their initial publications on frontline aggressive surgery for RPS,^16,17^ Bonvalot and Gronchi published surgical data demonstrating the safety of multivisceral resections.^14^ They showed that surgical morbidity was more frequent when high-risk structures such as major vessels or the pancreas were resected. In addition, they identified a correlation between morbidity and the number of resected organs, which was also observed in the present study. Later, a retrospective analysis of pooled data from the TARPSWG found no association between neoadjuvant chemotherapy or radiotherapy and surgical morbidity but did not explore this correlation in detail.^9^

Two recent studies demonstrated that the CCI is a more accurate predictor of the length and cost of hospital stay for RPS surgery^12,18^ than the Clavien–Dindo classification, replicating findings from the original CCI publication.^11^ While in these and other studies, the CCI has been used as a predictor to model different outcomes, we are aware of only a few studies that defined the CCI as the primary outcome and explored statistical models to predict the CCI.^19,20^ One difficulty lies in the specific distribution of this outcome variable, with observations concentrated in clusters at 0, 100, and in-between. Analyzing this distribution using ordinary regression models based on normal or other downward/upward-open distributions can lead to distorted estimators and reduced statistical power. Previous studies used a simplification by modeling only severe complications defined as a CCI ≥ 40,^19,20^ omitting parts of the information contained in the CCI. Here, we propose the use of zero-one inflated beta regression models.^21^ These models allow for simultaneous modeling of the three components of the CCI and provide a more accurate description of the outcome distribution. To the best of our knowledge, this is the first published study on a multivariable model of CCI.

Several conclusions can be drawn from our analyses, which merit further discussion. First, we found no influence of preoperative radiotherapy or chemotherapy combined with RHT, although both therapies were associated with higher CCI values in univariate analysis. In contrast, both the number of resected organs and intraoperative transfusion requirements independently predicted postoperative morbidity. Briefly, it is the extent and safety of surgery, not the intensity of neoadjuvant treatment, that determines postoperative morbidity. These results confirm previous findings^9,14^ in a large single-center cohort with a high frequency of neoadjuvant chemotherapy (31% vs 15%^9^ or ≥ 31%^14^) and radiotherapy (19% vs 23%^9^ or ≥ 14%^14^). The fact that neoadjuvant protocols are indicated on the basis of the risk of disease progression and recurrence suggests that increased morbidity in some patients reflects the aggressiveness of the disease, leading to more extensive surgery, rather than the toxicity of nonsurgical treatment.

Second, one new insight from the present study is that adding hyperthermia to neoadjuvant treatment is just as safe from a surgical point of view, even when followed by radiotherapy. With the role of neoadjuvant treatments in soft-tissue sarcoma being redefined in recent^22,23^ and ongoing (NCT04031677) studies, it is crucial to recognize that these treatments do not appear to influence perioperative morbidity. Their use, including intensive protocols, should not be limited by such concerns.

Third, we found no significant CCI increase in recurrent patients compared with primary resections, and recurrence did not change the association between preoperative treatments and morbidity, except for the combination of chemotherapy and radiotherapy. While the latter finding could be explained by the potentially larger field of irradiation in primary tumors, it should be interpreted cautiously since it is based only on a very small number of cases. Nevertheless, it has been shown that locally recurrent tumors tend to recur locally in the future,^24,25^ which makes them candidates for repeated resections. In high-risk situations, these patients may be considered for multimodal treatments, particularly if they had been offered upfront surgery previously.^26^ On the basis of our findings, multimodal treatments appear safe from a surgical perspective in these challenging situations.

Regional hyperthermia added to neoadjuvant chemotherapy has been shown to prolong local recurrence-free, disease-free, and overall survival in high-risk soft-tissue sarcomas.^6^ Since it has been implemented in only a limited number of expert centers, the generalizability of our conclusions might be questioned. However, given that the primary focus of this study was safety rather than efficacy, it is essential to acknowledge that the intensity of multimodal treatment likely did not contribute to increased morbidity. Other less intensive treatment strategies currently being investigated in ongoing trials, such as STRASS 2,^5^ are expected to demonstrate similar or better safety profiles.

## Conclusions

Postoperative morbidity in surgery for primary and recurrent RPS is related to the extent of surgery, not to neoadjuvant treatments. Intensive multimodal treatments, including the addition of regional hyperthermia to neoadjuvant systemic therapy, are safe from a surgical perspective.

## Supplementary Information

Below is the link to the electronic supplementary material.

Supplementary file1 (DOCX 854 kb)
