# Correction: Surgery for Retroperitoneal Soft Tissue Sarcoma is Safe Following Multimodal Treatment with Regional Hyperthermia

**DOI:** 10.1245/s10434-025-18730-7

**Published:** 2025-11-20

**Authors:** Mathilda Knoblauch, Cathy Werdel, Yanik Arlt, Lars H. Lindner, Dorit Di Gioia, Anton Burkhard-Meier, Luc Berclaz, Nina-Sophie Schmidt-Hegemann, Paul Schmidt, Jens Werner, Markus Albertsmeier

**Affiliations:** 1 Department of General, Visceral and Transplantation Surgery, Ludwig Maximilians Universität (LMU) Munich, LMU Hospital, Munich, Germany; 2 Department of Medical Oncology, Ludwig Maximilians Universität (LMU) Munich, LMU Hospital, Munich, Germany; 3 Department of Radiation Oncology, Ludwig Maximilians Universität (LMU) Munich, LMU Hospital, Munich, Germany; 4 Statistical Consulting for Science and Research, Berlin, Germany

**Correction to: Ann Surg Oncol (2025) 32:9116–9126** 10.1245/s10434-025-18141-8

In the published article, Tables 3 and 4 inadvertently omitted the modeled estimates of the Comprehensive Complication Index (CCI) by treatment group, which were included in the zero-inflated beta models as fixed effects. Table 3 has now been corrected to report the modeled probabilities of postoperative complications (CCI > 0) by treatment group, and Table 4 includes the expected CCI values (0 < CCI < 100) by treatment group. These corrections complete the presentation of the primary outcome but do not alter the study’s results or conclusions. The corrected Tables 3 and 4 are provided below.

**Table 3:** Influence of neoadjuvant therapy, gender, transfusion, age, number of resected organs and recurrence on CCI in the model examining the probability of experiencing any postoperative complication (CCI > 0)

	Risk CCI > 0 (Mean, 95% CI)
Neoadjuvant therapy	
No therapy (*n* = 155)	0.68 (0.57, 076)
Chemotherapy (*n* = 10)	0.80 (0.54, 0.95)
Radiotherapy (*n* = 17)	0.80 (0.61, 0.93)
Chemotherapy + Radiotherapy (*n* = 7)	0.50 (0.21, 0.79)
Chemotherapy + RHT (*n* = 36)	0.79 (0.65, 0.89)
Chemotherapy + RHT + radiotherapy (*n* = 36)	0.78 (0.64, 0.89)
*p* value	> 0.05 for all pairwise comparisons
Gender	
Female (*n* = 114)	0.73 (0.62, 0.82)
Male (*n* = 147)	0,72 (0.6, 0.81)
*p* value	0.77
Transfusion	
No (*n* = 132)	0.69 (0.58, 0.79)
Yes (*n* = 53)	0.83 (0.71, 0.91)
*p* value	**0.03**
Age (y)	
< 70	0.73 (0.63, 0.81)
≥ 70	0.70 (0.58, 0.80)
*p* value	0.49
Number of resected organs	
< 3	0.57 (0.44, 0.69)
≥ 3	0.79 (0.62, 0.88)
*p* value	**0.004**
Recurrence	
No	0.72 (0.62, 0.80)
Yes	0.74 (0.59, 0.86)
*p* value	0.65

RHT, regional hyperthermia

**Table 4:** Influence of neoadjuvant therapy, gender, transfusion, age, number of resected organs and recurrence on CCI in the model examining the probability of experiencing the severity of postoperative complication (0 < CCI < 100)

	Expected CCI (Mean, 95% CI)
Neoadjuvant therapy	
No therapy (*n* = 155)	30.2 (23.6, 37.7)
Chemotherapy (*n* = 10)	24.8 (14.5, 38.2)
Radiotherapy (*n* = 17)	32.7 (22.6, 45.1)
Chemotherapy + Radiotherapy (*n* = 7)	20.9 (9.9, 38.7)
Chemotherapy + RHT (*n* = 36)	36.1 (27.5, 46.0)
Chemotherapy + RHT + radiotherapy (*n* = 36)	37.7 (28.6, 47.7)
*p* value	> 0.05 for all pairwise comparisons
Gender	
Female (*n* = 114)	30.6 (24; 38.6)
Male (*n* = 147)	30.5 (23.8; 38.7)
*p* value	0.99
Transfusion	
No (*n* = 132)	29.3 (22.6, 37.1)
Yes (*n* = 53)	36.8 (28.9, 46.5)
*p* value	**0.03**
Age (y)	
< 70	30.2 (23.9, 38.0)
≥ 70	31.1 (23.9, 39.6)
*p* value	0.83
Number of resected organs	
< 3	22.2 (16.5, 29.3)
≥ 3	34.1 (26.4, 43.3)
*p* value	**0.002**
Recurrence	
No	30.5 (24.3, 38.2)
Yes	35.8 (25.9, 47.1)
*p* value	0.13

RHT, regional hyperthermia

Corresponding changes have been made to Supplemental Tables 3 and 4, which report the zero-inflated beta models for primary tumor patients, as well as to Tables 7 and 8, which report the models for recurrent tumor patients.

## Supplementary Information

Below is the link to the electronic supplementary material.

Supplementary file1 (DOCX 862 kb)

