# Supplementary material for: Correction: Surgery for Retroperitoneal Soft Tissue Sarcoma is Safe Following Multimodal Treatment with Regional Hyperthermia
Source: Ann Surg Oncol. 2025 Nov 20;33(2):1725–7. doi: 10.1245/s10434-025-18730-7 (PMC12765726; doi:10.1245/s10434-025-18730-7)
Supplement: Supplementary file 1 — Supplementary file1 (DOCX 862 kb) [file 10434_2025_18730_MOESM1_ESM.docx]

Supplemental materials

Primary tumours

**Table 1**: Baseline clinicopathologic characteristics of the study cohort (primary tumours)

| Characteristics | All  (n=153) |
| --- | --- |
| Sex, No. (%)  Female  Male | 61 (39.9)  92 (60.1) |
| Age, mean (+ SD), y | 60.1 (+13.0) |
| Grading, No. (%)  G1  G2  G3  Missing | 30 (19.6)  57 (37.3)  56 (37.3)  10 (86.5) |
| Blood transfusion (+SD), ml | 306.6 (+ 677.3) |
| R status, No. (%)  0  1  2  x  Missing | 92 (60.1)  119 (7.2)  11 (11.9)  1 (0.7)  3 (2) |
| Neoadjuvant radiation therapy, No. (%)  Yes  No  Missing | 56 (36.6)  94 (61.4)  3 (2.0) |
| Neoadjuvant chemotherapy, No. (%)  Yes  No  Missing | 87 (56.9)  64 (41.8)  2 (1.3) |
| Neoadjuvant hyperthermia, No (%)  Yes  No  Missing | 83 (54.2)  68 (44.4)  2 (1.3) |

**Table 2:** Postoperative Complications of the study cohort (primary tumours)

| Characteristics | All |
| --- | --- |
| The Comprehensive Complication Index, (+SD) | 23.73 (+23.39) |
| Clavien Dindo, No. (%)  Grade 0  Grade 1  Grade 2  Grade 3  Grade 4  Grade 5  Missing | 44 (28.8)  17 (11.1)  26 (17)  57 (37.3)  3 (2.0)  3 (2.0)  3 (2.0) |
| Mortality, No. (%)  Yes  No  Missing | 3 (2.0)  149 (97.4)  1 (0.7) |
| Reoperation, No. (%)  Yes  No | 33 (21.6)  120 (78.4) |
| Postoperative drainage, No. (%)  Yes  No | 24 (15.7)  129 (84.3) |

**Table 3:** Influence of neoadjuvant therapy, gender, transfusion, age, number of resected organs on CCI in the model examining the probability of experiencing any postoperative complication (CCI > 0) of the study cohort (primary tumours). RHT, regional hyperthermia.

|  | Risk CCI > 0 (Mean, 95% CI) |
| --- | --- |
| Neoadjuvant therapy |  |
| No therapy (n = 75) | 0.64 (0.48, 077) |
| Chemotherapy (n = 5) | 0.87 (0.46, 1.00) |
| Radiotherapy (n = 10) | 0.82 (0.55, 0.97) |
| Chemotherapy + Radiotherapy (n = 5) | 0.63 (0.25, 0.93) |
| Chemotherapy + RHT (n = 24) | 0.79 (0.58, 0.92) |
| Chemotherapy + RHT + radiotherapy (n = 29) | 0.76 (0.56, 0.90) |
| p-value | >0.05 for all pairwise comparisons |
| Gender |  |
| Female (n=58) | 0.75 (0.59, 0.86) |
| Male (n=90) | 0,74 (0.57, 0.85) |
| p-value | 0.88 |
| Transfusion |  |
| No (n=62) | 0.71 (0.53, 0.84) |
| Yes (n=33) | 0.87 (0.71, 0.96) |
| p-value | **0.05** |
| Age (y) |  |
| < 70 | 0.77 (0.63, 0.88) |
| > 70 | 0.70 (0.52, 0.83) |
| p-value | 0.9 |
| Number of resected organs |  |
| < 3 | 0.62 (0.43, 0.79) |
| > 3 | 0.79 (0.62, 0.90) |
| p-value | 0.1 |

**Figure 1**

**
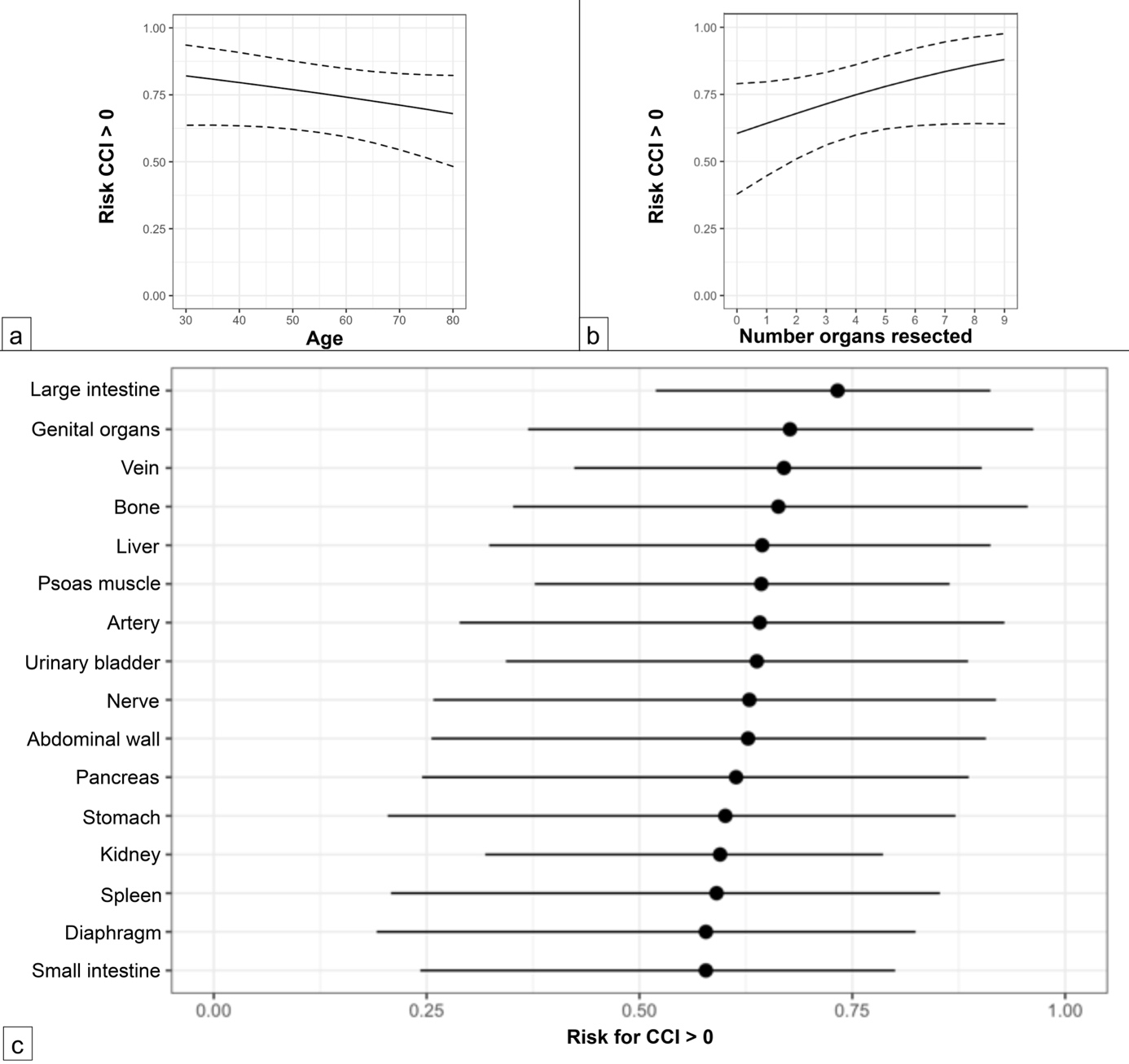
**

Influence of age (a), number of resected organs (b), recurrence (c) and certain resected organs (d) on CCI in the model examining the probability of experiencing any postoperative complication (CCI > 0) of the study cohort (primary tumours)

**Table 4:** Influence of neoadjuvnat therapy, gender, transfusion, age, number of resected organs and recurrence on CCI in the model examining the probability of experiencing the severity of postoperative complication (0 < CCI < 100) of the study cohort (primary tumours). RHT, regional hyperthermia.

|  | Expected CCI (Mean, 95% CI) |
| --- | --- |
| Neoadjuvant Therapy |  |
| No therapy (n = 75) | 26.1 (18.3, 35.8) |
| Chemotherapy (n = 5) | 25.1(12.7, 42.6) |
| Radiotherapy (n = 10) | 33.8 (21.3, 49) |
| Chemotherapy + Radiotherapy (n = 5) | 21.1 (9.4, 40.2) |
| Chemotherapy + RHT (n = 24) | 30.9 (21.0, 43.0) |
| Chemotherapy + RHT + radiotherapy (n = 29) | 32.7 (23.0, 45.1) |
| p-value | >0.05 for all pairwise comparisons |
| Gender |  |
| Female (n=58) | 28.6 (20.5; 38.6) |
| Male (n=90) | 28.3 (20.3.; 39.6) |
| p-value | 0.96 |
| Transfusion |  |
| No (n=62) | 26.2 (18.2, 36.4) |
| Yes (n=33) | 36.6 (26.7, 49.1) |
| p-value | **0.02** |
| Age (y) |  |
| < 70 | 29.8 (21.8, 40.5) |
| > 70 | 26.5 (18.6, 36.7) |
| p-value | 0.29 |
| Number of resected organs |  |
| < 3 | 23.8 (15.5, 34.8) |
| > 3 | 30.4 (21.8, 41.8) |
| p-value | 0.2 |

**Figure 2**

**
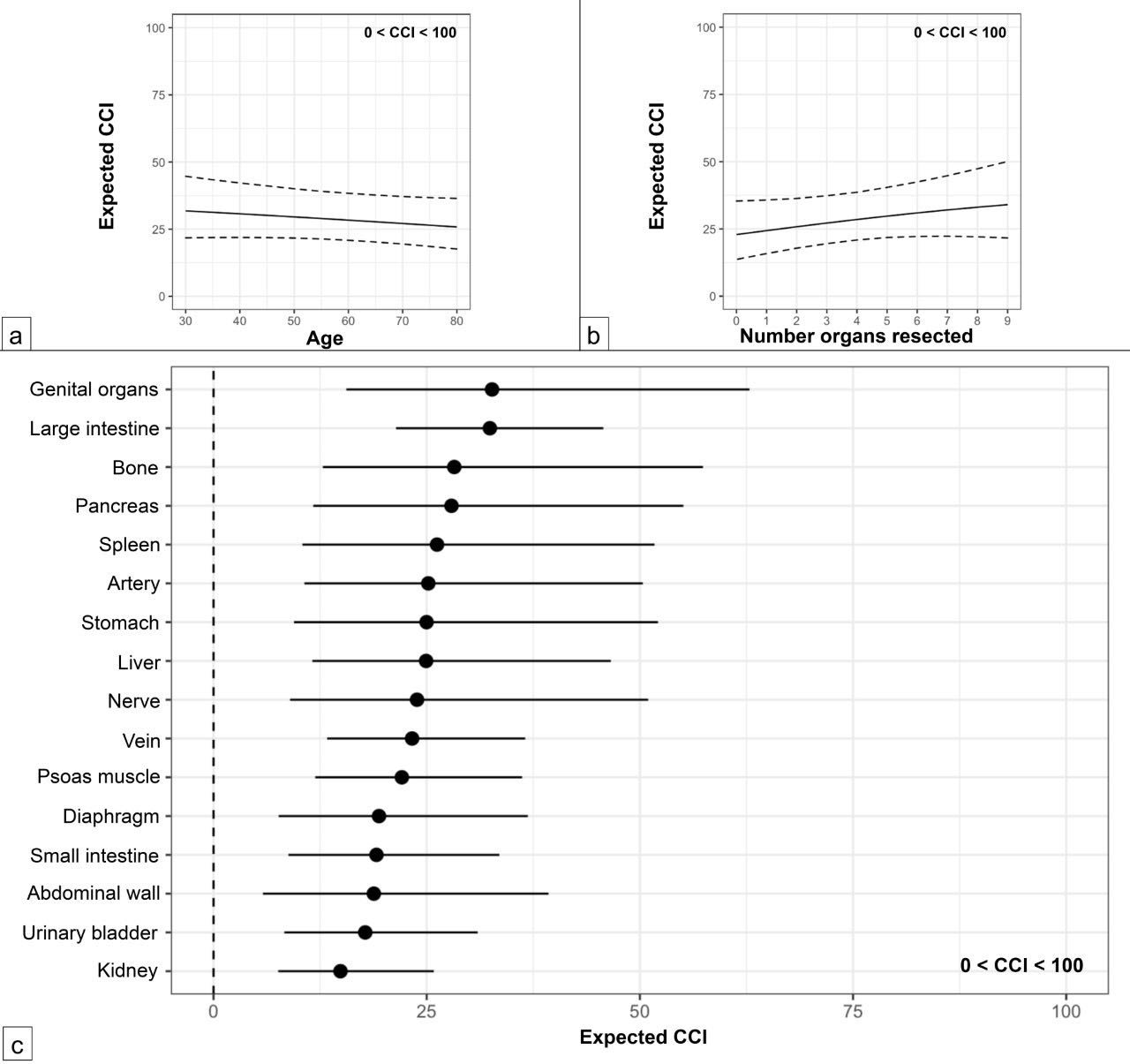
**

Influence of age (a), number of resected organs (b), recurrence (c) and certain resected organs (d) on CCI in the model examining the severity of complications (0 < CCI < 100) of the study cohort (primary tumours)

Recurrent tumours

**Table 5**: Baseline clinicopathologic characteristics of the study cohort (recurrent tumours)

| Characteristics | All  (n=182) |
| --- | --- |
| Sex, No. (%)  Female  Male | 91 (50.0)  91 (50.0) |
| Age, mean (+ SD), y | 58.2 (+12.6) |
| Grading, No. (%)  G1  G2  G3  Missing | 39 (21.4)  62 (34.1)  62 (34.1)  19 (10.4) |
| Blood transfusion (+SD), ml | 200.56 (+ 533.4) |
| R status, No. (%)  0  1  2  Missing | 67 (36.8)  73 (40.1)  29 (15.9)  13 (7.1) |
| Neoadjuvant radiation therapy, No. (%)  Yes  No  Missing | 34 (18.7)  130 (71.4)  18 (9.9) |
| Neoadjuvant chemotherapy, No. (%)  Yes  No  Missing | 40 (22)  138 (75.8)  4 (2.2) |
| Neoadjuvant hyperthermia, No (%)  Yes  No  Missing | 34 (18.7)  114 (62.6)  17 (9.3) |

**Table 6:** Postoperative Complications of the study cohort (recurrent tumours)

| Characteristics | All |
| --- | --- |
| The Comprehensive Complication Index, (+SD) | 26.67 (+30.15) |
| Clavien Dindo, No. (%)  Grade 0  Grade 1  Grade 2  Grade 3  Grade 4  Grade 5  Missing | 63 (34.6)  15 (8.2)  26 (14.3)  56 (30.8)  7 (3.8)  9 (4.9)  6 (3.3) |
| Mortality, No. (%)  Yes  No  Missing | 12 (6.6)  169 (92.9)  1 (0.5) |
| Reoperation, No. (%)  Yes  No  Missing | 44 (24.2)  130 (71.4)  8 (4.4) |
| Postoperative drainage, No. (%)  Yes  No  Missing | 54 (16.5)  146 (80.2)  6 (3.3) |

**Table 7:** Influence of neoadjuvant therapy, gender, transfusion, age, number of resected organs and recurrence on CCI in the model examining the probability of experiencing any postoperative complication (CCI > 0) of the study cohort (recurrent tumours). RHT, regional hyperthermia.

|  | Risk CCI > 0 (Mean, 95% CI) |
| --- | --- |
| Neoadjuvant therapy |  |
| No therapy (n = 80) | 0.72 (0.59, 0.81) |
| Chemotherapy (n = 5) | 0.72 (0.38, 0.94) |
| Radiotherapy (n = 7) | 0.76 (0.49, 0.95) |
| Chemotherapy + RHT (n = 12) | 0.72 (0.50, 0.89) |
| Chemotherapy + RHT + radiotherapy (n = 7) | 0.84 (0.60, 0.97) |
| p-value | > 0.05 for all pairwise comparisons |
| Gender |  |
| Female (n=54) | 0.77 (0.63, 0.88) |
| Male (n=57) | 0,72 (0.58, 0.84) |
| p-value | 0.42 |
| Transfusion |  |
| No (n=69) | 0.75 (0.61, 0.86) |
| Yes (n=19) | 0.83 (0.67, 0.95) |
| p-value | 0.32 |
| Age (y) |  |
| < 70 | 0.74 (0.6, 0.85) |
| > 70 | 0.76 (0.61, 0.87) |
| p-value | 0.76 |
| Number of resected organs |  |
| < 3 | 0.53 (0.34, 0.71) |
| > 3 | 0.84 (0.69, 0.93) |
| p-value | **0.002** |
| Recurrence |  |
| = 1 | 0.75 (0.62, 0.85) |
| > 1 | 0.78 (0.63, 0.89) |
| p-value | 0.53 |

**Figure 3**

**
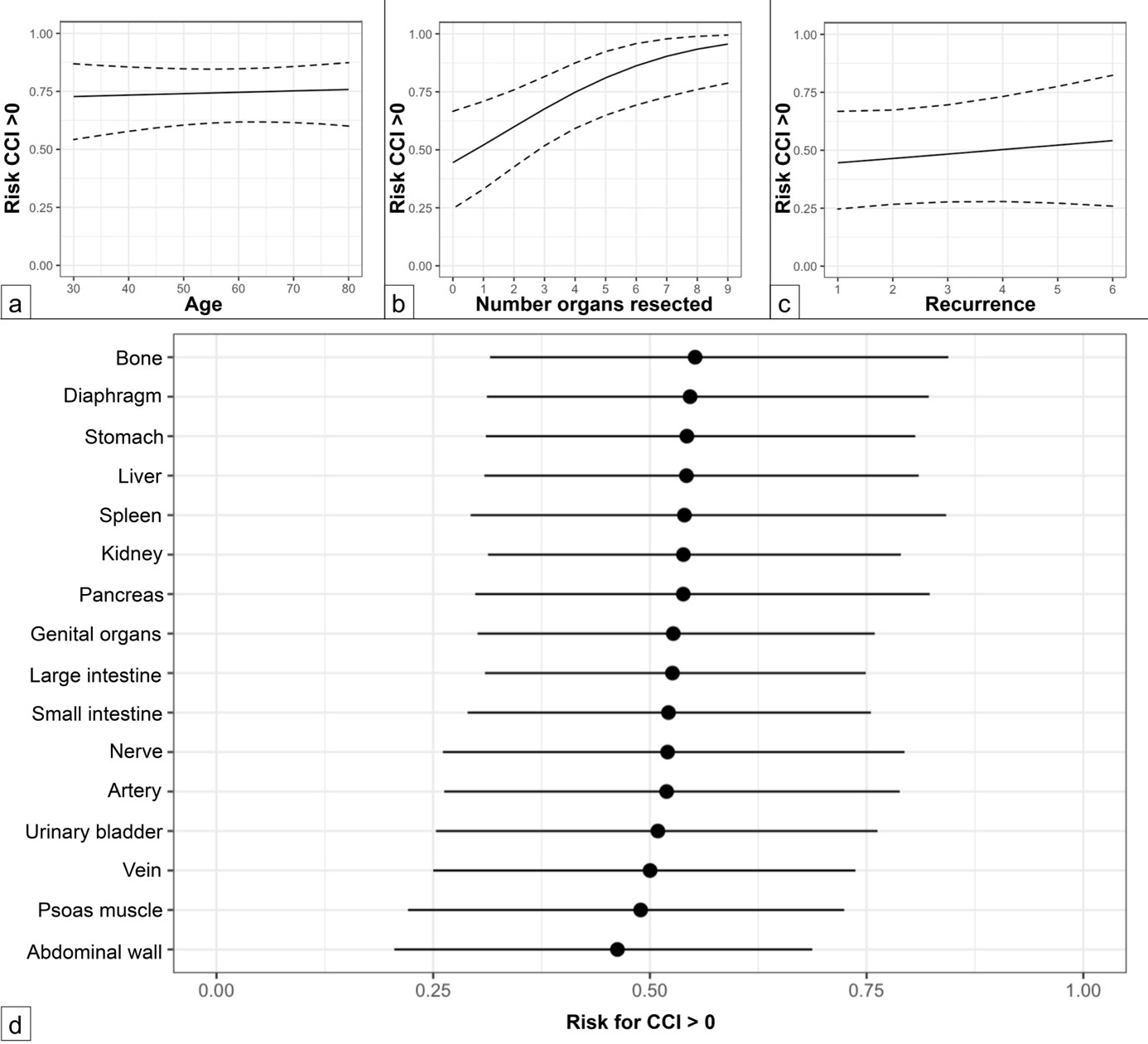
**

Influence of age (a), number of resected organs (b), recurrence (c) and certain resected organs (d) on CCI in the model examining the probability of experiencing any postoperative complication (CCI > 0) of the study cohort (recurrent tumours)

**Table 8:** Influence of gender, transfusion, age, number of resected organs and recurrence on CCI in the model examining the probability of experiencing the severity of postoperative complication (0 < CCI < 100) of the study cohort (recurrent tumours)

|  | Expected CCI (Mean, 95% CI) |
| --- | --- |
| Neoadjuvant therapy |  |
| No therapy (n = 80) | 34.9 (25.0, 45.9) |
| Chemotherapy (n = 5) | 22.3 (9.0, 43.0) |
| Radiotherapy (n = 7) | 30.2 (15.7, 49.6) |
| Chemotherapy + RHT (n = 12) | 37.8 (23.9, 54.1) |
| Chemotherapy + RHT + radiotherapy (n = 7) | 49.6 (29.4, 69.7) |
| p-value | 0.042 for chemotherapy vs  chemotherapy + RHT + radiotherapy  > 0.05 for all other pairwise comparisons |
| Gender |  |
| Female (n=54) | 36.4 (25.7, 48.4) |
| Male (n=57) | 34.0 (24.1, 45.0) |
| p-value | 0.57 |
| Transfusion |  |
| No (n=69) | 35,7 (25.0, 47.1) |
| Yes (n=19) | 40.6 (27.9, 55.2) |
| Missing (n=23) |  |
| p-value | 0.43 |
| Age (y) |  |
| < 70 | 33.6 (24.1, 44.3) |
| > 70 | 37.4 (26.2, 49.6) |
| p-value | 0.37 |
| Number of resected organs |  |
| < 3 | 23.4 (15.0, 33.9) |
| > 3 | 40.2 (28.3, 53.4) |
| p-value | **0.008** |
| Recurrence |  |
| = 1 | 35.2 (25.7, 45.8) |
| > 1 | 38.9 (26.5, 52.1) |
| p-value | 0.50 |

**Figure 4**

**
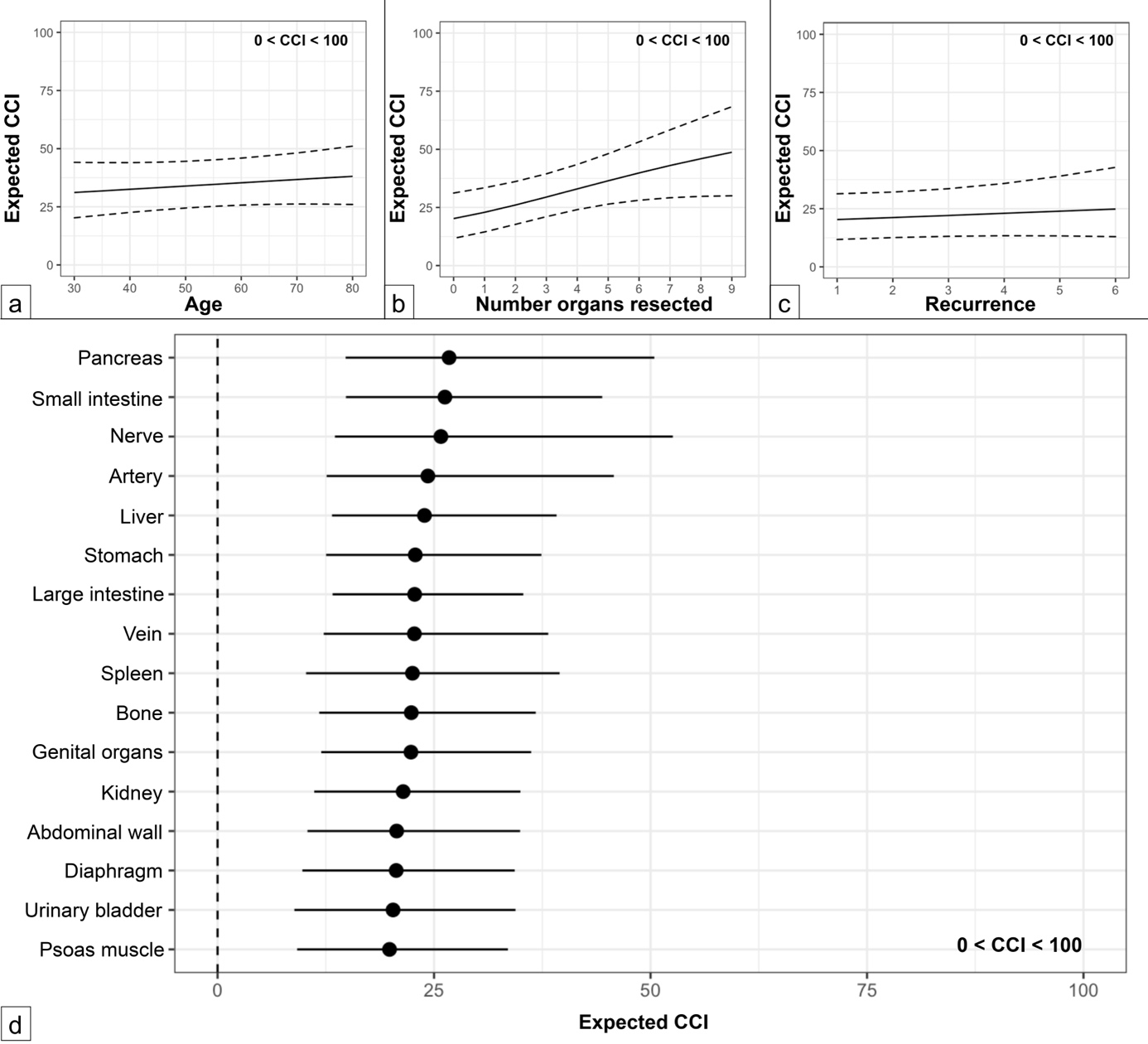
**

Influence of age (a), number of resected organs (b), recurrence (c) and certain resected organs (d) on CCI in the model examining the severity of complications (0 < CCI < 100) of the study cohort (recurrent tumours)
